# Informal care in times of a public health crisis: Objective burden, subjective burden and quality of life of caregivers in the Netherlands during the COVID‐19 pandemic

**DOI:** 10.1111/hsc.13975

**Published:** 2022-09-06

**Authors:** Leonoor Gräler, Leonarda Bremmers, Pieter Bakx, Job van Exel, Marianne van Bochove

**Affiliations:** ^1^ Erasmus School of Health Policy and Management Erasmus University Rotterdam Rotterdam The Netherlands; ^2^ Knowledge Centre for Governance of Urban Transitions De Haagse Hogeschool The Hague The Netherlands

**Keywords:** caregiver burden, COVID‐19, informal care, public health crisis, quality of life

## Abstract

In the Netherlands, about one‐third of the adult population provides unpaid care. Providing informal caregiving can be very straining in normal times, but the impact of a public health crisis on caregivers is largely unknown. This study focuses on the question of how caregiver burden changed following the COVID‐19 pandemic, and what characteristics were related to these changes. We use self‐reported data from a sample of 965 informal caregivers from the Netherlands 3 months into the pandemic to investigate how the objective burden (i.e. hours spent on caregiving) and the subjective burden had changed, and what their care‐related quality of life (CarerQol) was. We found that on average the subjective burden had increased slightly (from 4.75 to 5.04 on a 0–10 scale). However, our analysis revealed that some caregivers were more affected than others. Most affected caregivers were women, and those with low income, better physical health, decreased psychological health, childcare responsibilities, longer duration of caregiving and those caring for someone with decreased physical and psychological health. On average, time spent on care remained the same (a median of 15 h per week), but certain groups of caregivers did experience a change, being those caring for people in an institution and for people with a better psychological health before the pandemic. Furthermore, caregivers experiencing changes in objective burden did not have the same characteristics as those experiencing changes in perceived burden and quality of life. This shows that the consequences of a public health crisis on caregivers cannot be captured by a focus on either objective or subjective burden measures or quality of life alone. Long‐term care policies aiming to support caregivers to persevere during a future crisis should target caregivers at risk of increased subjective burden and a lower CarerQol, such as women, people with a low income and people with childcare responsibilities. Such policies should consider that reducing objective burden may not necessarily lead to a reduction in subjective burden for all caregivers*.*

**What is known about this topic**

- A substantial amount of care is provided by informal caregivers.
- Informal care can be burdensome, leading to negative consequences for the caregiver on various life domains.
- The COVID‐19 pandemic has had serious consequences for informal caregiving.

**What this paper adds**

- On average, informal caregivers in the Netherlands did not change the amount of time spent on caregiving. However, specific subgroups did report increased or decreased caregiving time.
- Subjective burden did increase. However, some subgroups experienced a larger increase and a larger decline in quality of life. These were not the same subgroups as those who experienced increases in caregiving time.
- This highlights the importance that researchers and policymakers account for heterogeneity among informal caregivers.

## 1 INTRODUCTION

Against the backdrop of rising health care expenditures, governments emphasise the need for informal care (Pavolini & Ranci, 2008). However, providing informal care can be very time consuming and perceived as burdensome (Bom et al., 2019). Increased caregiver burden has negative consequences for care recipients, sustainability of healthcare systems and societal costs. Burden of informal care can be exacerbated by events that increase stress factors or complicate routines. Such events can be changes in the health or the financial situation of the caregiver, changes in health of the care recipient, or an increase in other responsibilities (Pearlin et al., 1990). The COVID‐19 pandemic was a crisis in which many of these factors collided, affecting informal caregivers in many ways (Lorenz‐Dant & Comas‐Herrera, 2021). However, there may be differences among caregivers with regard to how they were affected. This has been understudied, while this knowledge is important to tailor interventions to support caregivers in times of a crisis.

In this paper, we will answer the following research questions: how did the burden of informal caregivers change following the COVID‐19 crisis? And how was this burden related to characteristics of caregivers, care recipients and the caregiving situation? We used self‐reported data to study to what extent caregivers experienced a change in burden 3 months into the COVID‐19 pandemic in the Netherlands. First, we explored which caregiver characteristics were related to a change in the number of hours spent on caregiving (i.e. objective burden). Subsequently, we analysed how characteristics related to changes in the perceived burden from caregiving (i.e. subjective burden). Lastly, we investigated care‐related quality of life during the pandemic. By combing objective burden, subjective burden and quality of life measures, it is not only possible to see which caregivers were most affected by the pandemic, but also the ways in which they were affected.

We contribute to the literature by identifying which characteristics relate to changes in objective and subjective burden during the COVID‐19 pandemic. Prior research shows that during the pandemic informal caregivers experienced changes in responsibilities as well as in mental, physical and financial health (de Sousa et al., 2022; Greaney et al., 2020; Lorenz‐Dant & Comas‐Herrera, 2021). These consequences differed for caregivers with different characteristics. Studies from various countries show that gender (Lorenz‐Dant & Comas‐Herrera, 2021; Raiber & Verbakel, 2021; Zwar et al., 2022), age (Budnick et al., 2021; Hofstaetter et al., 2022), employment (Truskinovsky et al., 2022), living situation of the care recipient (Prins et al., 2021; Smaling et al., 2022), relationship to the care recipient (Tur‐sinai et al., 2021) and network (Allen et al., 2022) were important characteristics that distinguish how caregivers were affected. Studies in the Netherlands found that there were differences between men and women, and between those in different relationships to the care recipient (Prins et al., 2021; Raiber & Verbakel, 2021; Smaling et al., 2022; Tur‐sinai et al., 2021) Previous literature thus shows that consequences differed across countries, which may be due to differences in measures, COVID impact and healthcare system (Lorenz‐Dant & Comas‐Herrera, 2021; Santini et al., 2022; Tur‐sinai et al., 2021). In the Netherlands, there were relative large increases in informal care and decreases in formal care compared to other countries (Tur‐sinai et al., 2021).

Despite the growing body of research on informal care during COVID, so far, no studies seem to have investigated how objective burden, subjective burden and quality of life were related during the pandemic, and whether this relationship differs between groups. Therefore, in this paper, we study the question: To what extent were informal caregivers affected during the COVID‐19 pandemic in the Netherlands, and to what extent did that differ between informal caregivers with different characteristics?

## 2 CONCEPTUAL MODEL

### 2.1 Caregiver burden measurement

Caregiver burden represents the overall consequences of caregiving (Pearlin et al., 1990). In this paper, we focus on objective burden, subjective burden and care‐related quality of life. The *objective burden* of care is the burden of care measured by the time spent on caregiving. However, time spent on care may not necessarily reflect how the caregiving burden is perceived (i.e. *subjective burden*) (Montgomery et al., 1985). According to the caregiver stress process model (Pearlin et al., 1990), subjective burden is the result of the emotional evaluation of aspects directly related to caregiving itself (e.g. needs of the care recipient, time spent on caregiving), which is mediated through aspects secondary to the care process such as difficulty combining caregiving with other activities and responsibilities, or economic strains. Furthermore, demographic factors, such as gender, and socioeconomic status (e.g. gender, socioeconomic status) influence both the time spent on caregiving, but also directly influence how caregiving is perceived. By combining measures of objective burden and subjective burden, groups who provide a lot of care and groups who experience a lot of burden can be distinguished.

Finally, subjective burden may not capture the overall impact of caregiving on all life domains relevant to caregivers. Quality of life is conceptually different from subjective burden (Chappell & Reid, 2002; Yates et al., 1999). It captures the effect of the appraisal of the caregiving situation on overall well‐being and is influenced by both aspects directly or indirectly related to caregiving and aspects beyond the caregiving process (Chappell & Reid, 2002). To measure care‐related quality of life, we use the CarerQol, which is a caregiver‐specific quality of life measure (Brouwer et al., 2006; Hoefman et al., 2013). The dimensions of this instrument consist of satisfaction, the relationship with the care recipient, psychological well‐being, ability to combine daily activities with care, financial well‐being, support network and physical well‐being. All dimensions are specifically asked in the context of informal caregiving. Care‐related quality of life is strongly associated with objective and subjective burden, but also encompasses a broader set of potentially relevant impacts of caregiving on the overall quality of life of caregivers.

### 2.2 Caregiver characteristics and COVID‐19

Based on the work of Pearlin et al. (1990), Yates et al. (1999) and Chappell and Reid (2002), we discuss characteristics associated with objective burden, subjective burden and care‐related quality of life that are relevant in the context of COVID‐19. COVID‐19 may have influenced both the characteristics as well as their relationship to the outcome. We distinguish three groups of characteristics: care recipient's need for care, caregivers' dispositional and restrictive characteristics and help from others. In this section, we will discuss the potential effects of the pandemic on caregivers based on these characteristics.

#### 2.2.1 Care recipient's need for care

Characteristics of the care recipient are related to variation in the demand for care and include the health of the care recipients and the nature of the condition (Chappell & Reid, 2002; Pearlin et al., 1990; Yates et al., 1999). Whereas the first two determine how much and what care is needed, the latter determines among whom the care is potentially divided. A health decline during the pandemic would increase the need for care. Furthermore, how caregivers experienced the pandemic may be related to the nature of the condition of the care recipient, because psychological conditions and physical conditions may have been experienced differently by caregivers. The changes in health during the pandemic and the nature of the condition before the pandemic are therefore expected to be related to the caregiver outcomes.

#### 2.2.2 Caregiver dispositional and restrictive characteristics

Demographic and socioeconomic characteristics are associated with caregiver outcomes (Chappell & Reid, 2002; Pearlin et al., 1990; Yates et al., 1999). The pandemic may have influenced the relationship between these characteristics and the consequences of caregiving. For example older caregivers may have felt more at risk. This may also be the case for the relationship between health of the caregiver and outcomes. Caregivers who were already struggling with their health may have been less inclined to care or experienced more stress, because of their own health risk. Furthermore, the psychological and physical health of caregivers may have changed during the pandemic (Park, 2020).

Another dispositional characteristic is the relationship between the caregiver and care recipient, because it implies certain expectations with regard to caregiving (Fletcher, 2020). This includes the type of the relationship and its duration (Chappell & Reid, 2002; Pearlin et al., 1990; Yates et al., 1999). Both affirm the obligation and willingness to care felt within a relationship, which may be related to changes in caregiving tasks during the pandemic. For instance, most informal caregivers who stopped proving care, provided care for less than 10 h a week on average (Rodrigues et al., 2021). However, those with strong ties often provide more intensive informal care (de Boer et al., 2020). Therefore, we expect that the relationship between the caregiver and care recipient is of importance in how the pandemic was experienced and whether informal caregivers changed the amount of time spent on caregiving.

Whether the informal caregiver lives together with the care recipient (De Boer et al., 2020) and the travel distance (White, 2020) may have also had an influence, because contact with people from outside the household was restricted and care homes were closed for visitors (De Boer et al., 2020). This made it difficult to provide care outside the household, while within the household it was difficult to escape the caregiving situation.

Finally, caregiver outcomes are related to responsibilities such as work or childcare. These other responsibilities not only influence the time available for caregiving, but also how caregiving is experienced (Pearlin et al., 1990). Combining work and caregiving could have become more complicated during the COVID‐19 crisis (Lafferty et al., 2021). Furthermore, time spent on childcare may have intensified due to school closures and home schooling. We expect that caregivers with children experienced an increased subjective burden, as prior studies show that childcare responsibilities affect subjective burden (Koopman et al., 2020). To conclude, we expect that the impact of the pandemic on other responsibilities of caregivers may have influenced the time spent on caregiving and the perceived burden of caregivers.

#### 2.2.3 Help from others

How the care needs of the care recipient are fulfilled, depends, among other things, on the social network of the care recipient (Broese van Groenou & De Boer, 2016). The availability of a social network is thus of importance for spreading the burden among caregivers, resulting in lower burden. Furthermore, the number of potential caregivers may have changed because of the pandemic (Rodrigues et al., 2021), which could have resulted in changes in burden and quality of life.

## 3 DATA AND METHODS

### 3.1 Data sample

We collected data through a questionnaire which we developed based on validated measures (Hoefman et al., 2013). The questionnaire was administered online by a commercial agency with a large panel in The Netherlands. The agency asked members of their panel aged 18 years and older whether they were informal caregiver for someone of 18 years and older for at least 3 months (although we only included caregivers providing care for more than a month before the start of the pandemic). In this message to panel members, informal care was described as giving help or providing care to someone, for example their partner, a family member or friend, because of a physical, mental or cognitive limitation or the consequences of ageing.

A total of 3116 members of were invited by the agency to participate in this study. Of them, 2485 (79.7%) clicked on the link to the survey that was provided in the invitation. After reading the information about the survey, the inclusion criteria and the informed consent form, 1006 members of the panel agreed to participate in the survey. After inspection of the data, 41 participants were excluded because they did not meet the inclusion criteria after all; they reported to provide care to a person younger than 18 years or were caregivers for less than 4 months, meaning they could not assess the situation before the pandemic. This resulted in a final sample for analysis of 965 participants.

**FIGURE 1:**
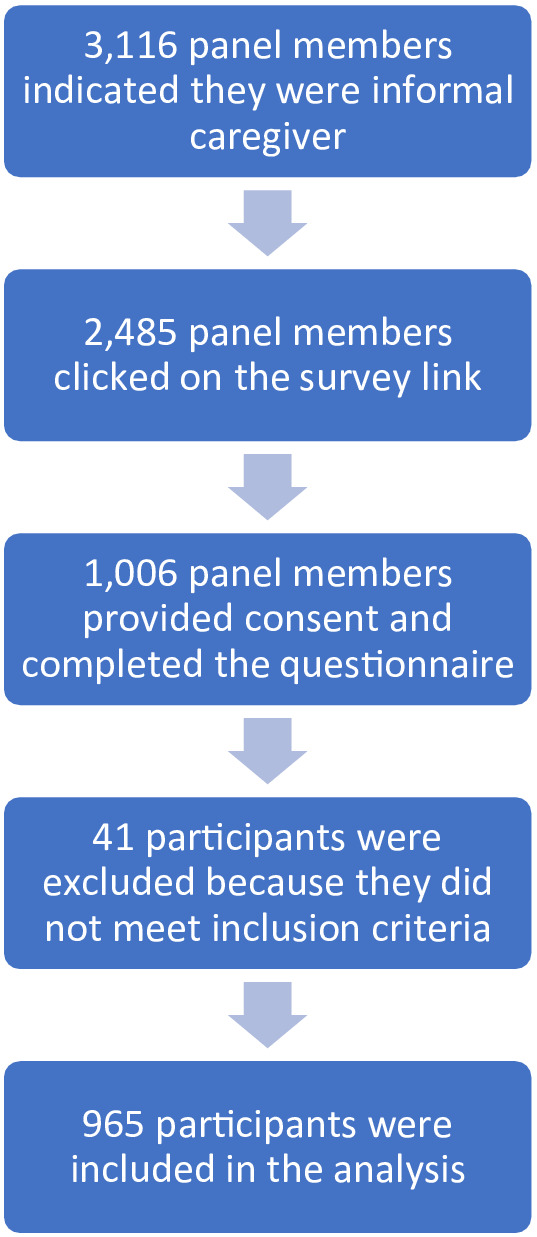
Participant identification and response.

Participants were instructed to keep in mind the person they provided care to while filling out the questionnaire. If they provided care to more than one care recipient, they were asked to keep in mind the one for whom the caregiving was most straining. This was done for reasons of feasibility in regards to questions about the socio‐demographic characteristics, health and care needs of the care recipient and their relationship. 68.9% of the respondents indicated that they provided care to only one person, 23.3% to two persons and 7.8% to three or more persons. Respondents were not allowed to skip questions in the online questionnaire, therefore, there were no missing data points. Data and STATA code are available upon request (Figure 1).

### 3.2 Ethics

Participants were informed about the topic and aims of the study and the data collection and provided informed consent before filling out the questionnaire.

### 3.3 Timing of the survey

The questionnaire was administered between June 2 and 16, 2020. At that point, a lockdown to prevent further spread of COVID‐19 in the Netherlands had been in place for almost 3 months. People were urged to keep 1.5 m distance from people that were not in their household, stay at home as much as possible and minimise unnecessary travelling (Dutch Government, 2020). Many formal care providers scaled down their usual care to essential care. Daycare facilities were closed and home care was regularly suspended, among other things (Dutch Ministry of Health, 2020). In addition, many people declined formal care due to concerns regarding COVID‐19 infection and personnel shortage for more urgent care. The Oxford stringency index (Hale et al., 2021), which indices the response of government to the pandemic, had been around 79 throughout April and May, and was 63 during the period of data collection because in the aftermath of the first wave of infections, some measures had been relaxed (Dutch Government, 2020). For example, children under the age of 12 started to be able to go to school or daycare again for a few days per week, where before the schools were fully closed. Also, care organisations in regions that did not have many confirmed COVID‐19 cases returned to care as usual. In some nursing homes, one designated family member was allowed to visit their family member again, although still under very restricted circumstances.

### 3.4 Outcome variables

We report three main outcome variables: the changes in (1) objective burden and (2) subjective burden between the time of the survey and the situation prior to the COVID‐19 measures and (3) the care‐related quality of life of informal caregivers at the time of the survey. Care‐related quality of life was not measured retrospectively, because of concerns about the length and complexity of the CarerQol questionnaire. An overview of how all variables were measured and constructed is included in Appendix S1.

The objective burden of care was measured as the sum of hours spent on household tasks, personal care, practical support and emotional support in the past week, and during a regular week before the start of the pandemic. The difference between these two values was used in this study.

The subjective burden of care was measured using a self‐rated burden scale (Van Exel et al., 2004). That is participants were presented with a visual analogue scale ranging from 0 to 10 (0 = not straining at all, 10 = much too straining) and asked to indicate how burdensome the caregiving situation was in the past week, and how burdensome it was during a regular week before the start of the pandemic. The difference between the two values was used in this study.

The CarerQol (Brouwer et al., 2006) consists of seven items addressing the potential impacts of caregiving on the quality of life of caregivers, of which two concern positive and five concern negative impacts, with three answering categories each. Using utility weights developed by Hoefman et al. (2014), a care‐related quality of life score was computed that ranges from 0 to 100, with 100 defined as the highest possible care‐related quality of life and 0 as the worst possible care‐related quality of life.

### 3.5 Independent variables

We included characteristics that may be related to changes in objective or subjective burden and to care‐related quality of life during the pandemic based on the literature discussed in Section 2.2.

The care recipient's health was assessed by the caregiver on scales from 1 to 10, with worse health indicating a higher need for care. The change in physical and mental health was used to assess whether increasing health problems during the pandemic affected the need. The physical and mental health before the pandemic were used to assess the nature of the pre‐existing health condition.

We included the caregiver's dispositional or restrictive characteristics gender, age, highest attained education, financial status (i.e. ability to make ends meet) and physical and mental health (measured in the same way as for the care recipient). In addition, we asked about other responsibilities of the caregiver, including work status, time spent in paid work during and before the pandemic and childcare responsibilities. Aspects of the caregiving situation included the type of their relationship, the duration of caregiving, whether they shared a household and, if not, the living situation of the care recipient and the travel distance (in minutes) to where the care recipient lives.

The variable ‘social network’ assessed the network of the care recipient, as a resource for sharing or (temporarily) taking over the care and as a source of emotional support. In the survey, we asked the informal caregiver to estimate how many other people the care recipient could ask for help, if needed. We also collected data on formal care use by the care recipient, which may be a substitute for informal care. We do, however, not use these data because most informal caregivers indicated not to know enough about how much and which formal care the care recipient received.

### 3.6 Methods

For objective and subjective burden, we regress the change in these variables between the time of the survey and the situation prior to the pandemic on the care need of the care recipient, the characteristics of the caregiver and the social network. We do the same for the level of care‐related quality‐of‐life during the pandemic. We use ordinary least squares regressions with standard errors clustered at individual level. To show the possible mediator effects of objective burden and subjective burden, Table S1 in Appendix 8.2 provides additional analyses. Also, to show the heterogeneity underneath the mean effect of variables on the change in objective and subjective burden, a multinomial logistic regression was performed. These analyses are included in Appendix 8.2, Tables S2 and S3. Results of these analyses are discussed and compared to the analyses in the results in the Appendix S1. We do not interpret the coefficients as causal effects. Instead, we are interested in the magnitudes and direction of the associations because this helps to understand how the burden and the care‐related quality of life changed for caregivers with different characteristics in the first phase of the pandemic.

## 4 RESULTS

### 4.1 Descriptive statistics

Table 1 presents the descriptive statistics of the three outcome variables and the main characteristics of the sample, which consisted of 965 caregivers.

**TABLE 1:** Descriptive statistics

	Before COVID‐19 (*t* = 0)		During COVID‐19 (*t* = 1)		Change	
	% or Mean	(SD)	% or Mean	(SD)	% or Mean	(SD)
Objective burden						
Number of hours of care	24.79	(27.79)	24.74	(27.53)	0.05	(5.84)
Subjective burden						
Perceived burden of the situation	4.75	(2.45)	5.04	(2.55)	0.30	(1.37)
Care‐related quality of life						
CarerQol			76.27	(18.40)		
Care recipient care need						
Psychological health	7.46	(1.97)	6.93	(2.11)	−0.52	(1.41)
Physical health	6.70	(1.84)	6.40	(1.91)	−0.31	(1.30)
Caregiver characteristics						
Woman (=1)			55			
Age			52.20	(15.97)		
Level of education						
Low			18			
Middle			44			
High			38			
Ability to make ends meet						
Very difficult			8			
Somewhat difficult			37			
Somewhat easy			42			
Very easy			13			
Psychological health	7.56	(1.70)	7.35	(1.83)	−0.22	(1.24)
Physical health	7.20	(1.62)	7.03	(1.67)	−0.16	(0.95)
Employment status						
Working			57			
Not working			20			
Retired			24			
Hours employment	18.46	(17.38)	17.19	(17.10)	−1.27	(6.22)
Childcare responsibilities			23			
Relationship to respondent						
Partner			23			
Parent			39			
Other family member			22			
Friends and other			16			
Duration of care			7.27	(7.69)		
Living situation						
With respondent			28			
Other private home			54			
Nursing or care home			18			
Travel distance			19.92	(36.79)		
Social network			2.52	(1.46)		

**FIGURE 2:**
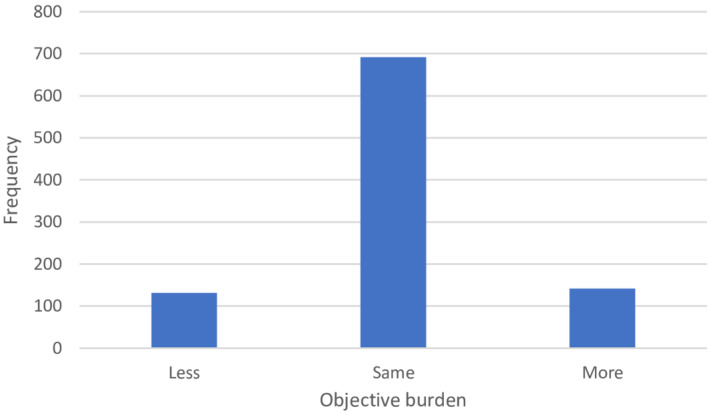
The hours spent on caregiving during the pandemic, compared to before the pandemic.

The mean time spent on caregiving was 24.79 h per week before the pandemic and 24.74 h per week during the pandemic. However, the distribution was heavily skewed: the median was 15 h per week, both before and during the pandemic. The study sample consisted of caregivers who provide rather intensive informal care, compared to of informal caregivers in general. The time spent on care by informal caregivers in a large Dutch sample was 7.4 h per week, with a median of 3 h per week (de Boer et al., 2020). Six hundred and ninety‐two out of 965 informal caregivers indicated they experienced no change, and continued to provide the same amount of care despite of the pandemic (Figure 2). However, the standard deviation in the changes was 5.84 h, pointing to considerable heterogeneity.

**FIGURE 3:**
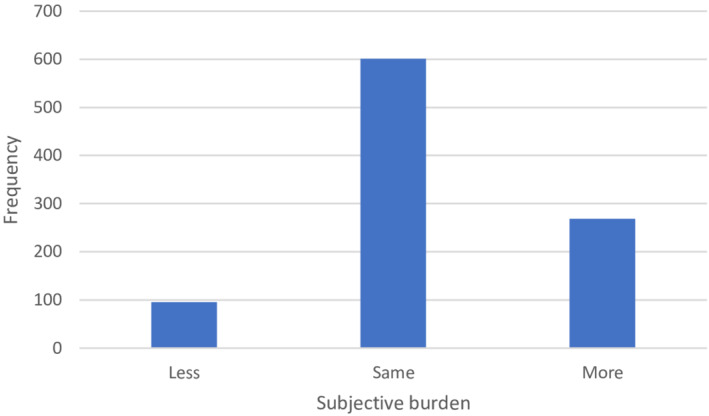
The subjective burden experienced during the pandemic, compared to before the pandemic.

The subjective burden was around 4.75 on a scale from 0 to 10 before the pandemic, and 5.04 during the pandemic. There was a statistically significant (*p* < 0.05) increase of 0.30 points in subjective burden. Again, the standard deviation of 1.37 reveals considerable heterogeneity in the changes. In a large sample from the Netherlands, 9.1% of caregivers were heavily burdened (de Boer et al., 2020). If scores of 8 and higher are considered as being heavily burdened, 12.0% of caregivers felt heavily burdened before the pandemic in our sample. During the pandemic, that percentage increased to 17.5%. Six hundred and one out of 965 informal caregivers did not experience any change (Figure 3).

**FIGURE 4:**
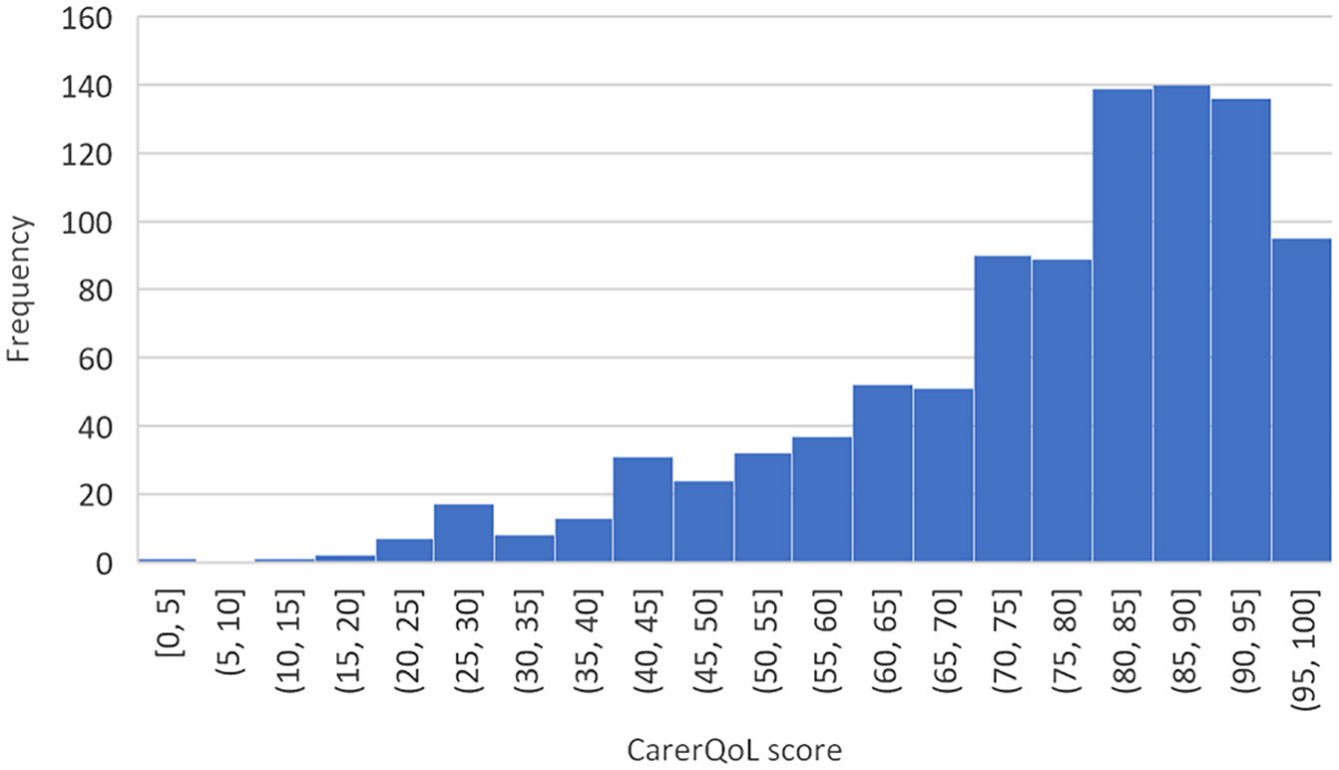
The frequency of CarerQol values during the pandemic. Lowest possible score is 0 and maximum score is 100.

The mean quality of life during the pandemic was 76.27 on a scale from 0 to 100. However, there is considerable heterogeneity in the group (Figure 4).

The changes in objective and subjective burden of care were positively but weakly correlated (0.08). The changes in objective and subjective burden of care were both weakly negatively correlated with CarerQol scores (−0.02 and −0.08). These measures apparently seem to capture different aspects of the impact of informal caregiving.

### 4.2 Regression analyses

In Table 2, we report our analyses of the three outcome variables by three groups of characteristics: the care need of the care recipient, dispositional and restrictive characteristics of the caregiver and social network.

**TABLE 2:** The outcomes regressed on the characteristics

	△ Objective burden		△ Subjective burden		Care‐related quality of life (*t* = 1)	
	*β*	(SE)	*β*	(SE)	*β*	(SE)
Care recipient care need						
Psychological health of recipient (*t* = 0)	0.295^**^	(0.117)	−0.002	(0.027)	0.907^***^	(0.308)
∆ Psychological health of recipient	0.001	(0.152)	−0.107^***^	(0.035)	−0.027	(0.401)
Physical health of recipient (*t* = 0)	0.086	(0.126)	−0.016	(0.029)	−0.463	(0.332)
∆ Physical health of recipient	−0.145	(0.166)	−0.178^***^	(0.038)	−0.316	(0.439)
Caregiver characteristics						
Woman (=1)	0.289	(0.423)	0.319^***^	(0.096)	−1.482	(1.117)
Age caregiver	−0.012	(0.018)	0.005	(0.004)	0.147^***^	(0.047)
Education (ref = low)						
Middle	0.182	(0.536)	−0.045	(0.122)	2.941**	(1.414)
High	0.390	(0.575)	0.022	(0.131)	−0.007	(1.518)
Ability to make ends meet (ref = fairly easily)						
With great difficulty	−0.500	(0.790)	0.107	(0.180)	−12.407***	(2.086)
With some difficulty	−0.690	(0.436)	0.232**	(0.099)	−3.226***	(1.151)
Easily	−0.360	(0.596)	0.167	(0.136)	1.427	(1.574)
Psychological health of caregiver (*t* = 0)	−0.000	(0.159)	−0.058	(0.036)	2.435***	(0.420)
∆ Psychological health of caregiver	0.042	(0.178)	−0.136***	(0.040)	1.842***	(0.469)
Physical health of caregiver (*t* = 0)	0.007	(0.160)	0.103***	(0.037)	1.400***	(0.424)
∆ Physical health of caregiver	0.178	(0.227)	0.059	(0.052)	1.887***	(0.598)
Work status (ref = working)						
Not working	−0.890	(0.868)	0.048	(0.198)	−3.218	(2.291)
Retired	−0.920	(0.909)	−0.026	(0.207)	−3.785	(2.401)
Hours employment (*t* = 0)	−0.012	(0.025)	0.002	(0.006)	−0.108*	(0.065)
∆ Hours employment	−0.051	(0.037)	−0.013	(0.009)	−0.100	(0.099)
Childcare responsibilities (*t* = 1)	−0.254	(0.516)	0.211*	(0.118)	−6.298***	(1.362)
Relationship (ref = partner)						
Parent	0.072	(0.725)	−0.038	(0.165)	2.277	(1.915)
Other family member	0.601	(0.751)	−0.094	(0.171)	1.398	(1.982)
Friends and other	0.785	(0.833)	−0.095	(0.190)	3.481	(2.199)
Duration of care	0.038	(0.026)	0.014**	(0.006)	−0.143**	(0.069)
Living situation (ref = in same home)						
Other private home	−1.095	(0.675)	0.189	(0.154)	1.996	(1.782)
An institution	−3.108***	(0.747)	0.153	(0.170)	−1.005	(1.974)
Travel distance	0.001	(0.005)	−0.001	(0.001)	−0.043***	(0.014)
Social network of recipient	−0.060	(0.135)	0.033	(0.031)	1.636***	(0.356)
Constant	−0.666	(1.815)	−0.544	(0.414)	36.994***	(4.793)
Observations	965		965		965	
*R* ^2^	0.055		0.111		0.336	

* *p* < 0.10

** *p* < 0.05

*** *p* < 0.01.

#### 4.2.1 Change in objective burden

In terms of care need of the care recipient, a 1‐point better mental health of the care recipient before the pandemic was associated with an 18‐min larger increase in caregiving (0.295 h). Furthermore, the variable living situation was significantly related to changes in objective burden. Caregivers who provided care to someone who lives in an institution reported a larger decrease (−3.108 h) in the objective burden than the reference category caring for someone living in the same household.

#### 4.2.2 Change in subjective burden

A decrease in physical or mental health of the care recipient during the pandemic was associated with increased subjective burden. Furthermore, caregivers who had difficulty making ends meet reported a larger increase in subjective burden than the reference group, as did women, people with childcare responsibilities and caregivers who were in better physical health before the pandemic. In all cases, the magnitude of the coefficient was small compared to the standard deviation in subjective burden. Furthermore, for caregivers who provided care to someone living in an institution, the change in objective burden was not accompanied by a change in subjective burden. These caregivers did not report a significantly different quality of life score than caregivers providing care for someone living in the same household. However, the duration of care was positively related to an increase in caregiver burden, even though we controlled for the physical and mental health of the care recipient.

#### 4.2.3 Quality of life

Care‐related quality of life was positively associated with age. A 1‐year increase in age was associated with 0.15 points increase on care‐related quality of life on a scale from 0 to 100. Having a middle level of education also had a positive relationship to quality of life compared to having a low level of education. Furthermore, psychological and physical health state before the pandemic and changes therein were positively related to the quality of life. Finally, respondents reporting some or great difficulty making ends meet indicated a 3.2‐point and 12.4‐point lower care‐related quality of life, respectively, as compared to those who answered ‘fairly easily’. Childcare responsibilities and being retired were related to 6.3‐point and 3.8‐point lower care‐related quality of life respectively. Furthermore, duration of care and travel distance were negatively associated with care‐related quality of life. The mental health of the care recipient before the pandemic was significantly positively associated with the care‐related quality of life. Also, for every extra person in the social network of the recipient, the quality of life of the caregiver was 1.6 points higher. Additional analyses (see Appendix 8.2.1) furthermore showed that the changes in objective and subjective burden are negatively associated with care‐related quality of life.

## 5 DISCUSSION

In this paper, we highlight which groups of caregivers—and indirectly, care recipients—were particularly vulnerable to a public health crisis such as the COVID‐19 pandemic and quantified the differences between these groups. Our research contributes to understanding how the changes in circumstances related to changes in informal care burden and quality of life. This information helps tailoring policy to support caregivers to those who need it the most. It also highlights which informal caregivers may be vulnerable for personal crises, which occur on a much smaller scale but a more regular basis. After all, disruptions in the caregiving process, such as changing responsibilities or loss of income, are not unique to the pandemic.

### 5.1 Main findings

We report three main findings. First, on average the time spent on caregiving did not change, while the subjective burden increased slightly. This finding suggests that (1) the subjective burden is also related to other aspects than the time spent on caregiving and that (2) the pandemic was associated with an increase in the subjective burden which cannot be directly linked to an increase in caregiving hours. The change in the subjective burden, however, is rather small. This is in line with findings from Austria that there was no change in the objective burden (Rodrigues et al., 2021), and with studies from Australia, Portugal, the United States, Argentina, Canada, India, Italy, Japan, Taiwan, Germany and the United Kingdom, that report an increase in subjective burden (de Sousa et al., 2022; Hofstaetter et al., 2022; Lorenz‐Dant & Comas‐Herrera, 2021; Truskinovsky et al., 2022). Caregivers experienced not only negative effects, but also positive effects of the pandemic, such as the slower pace (Lightfoot et al., 2021). This could contribute to explaining why the subjective burden changed only slightly during the COVID‐19 pandemic.

Second, there were considerable differences between subgroups of informal caregivers in the changes in objective and subjective burden and care‐related quality of life during the pandemic. The ability to continue providing care during the pandemic depended on the living situation of the care recipient, with larger declines in objective burden among caregivers providing care to someone living in a nursing home. Also, a better mental health of the care recipient before the pandemic was positively related to the change in hours of care provided. In terms of subjective burden, the following caregivers experienced an increase: caregivers having difficulty to make ends meet, women, those with childcare responsibilities, those with better physical health before the pandemic, those who had been caring for a longer period and caregivers who provided care for someone with declining mental and physical health. Similarly, Lorenz‐Dant and Comas‐Herrera (2021) reported that in countries such as Italy, the United Kingdom and Australia the risk of increased burden was greater among women, younger caregivers and caregivers with financial difficulties. Additional analyses (see Appendix 8.2.1) showed that an increase in objective and subjective burden was related to lower care‐related quality of life.

Third, the characteristics related to a change in objective burden were not necessarily the same as for changes in subjective burden or care‐related quality of life. Groups of caregivers who increased caregiving during the pandemic did not all report increased subjective burden or a lower quality of life, vice versa. For example a larger social network does not seem to be related to changes in objective and subjective burden, but is related to higher care‐related quality of life. Also, while men and women did not differ on changes in time spent on caregiving, women experienced a larger increase in subjective burden and a lower care‐related quality of life. In general, women experienced more negative well‐being consequences from the pandemic (Rodrigues et al., 2021), although other research shows that the gender gap in well‐being of caregivers may be decreasing (Raiber & Verbakel, 2021). Our results could thus partly be due to a more general negative effect of the pandemic on women. Mechanisms behind this should be studied in future research. Policy aimed at supporting caregivers should account for the different drivers of objective and subjective burden and quality of life between caregivers.

Changes in objective burden, subjective burden and quality of life are thus explained by characteristics of the caregiver, care recipient and their relationship that are also featured in former work (Chappell & Reid, 2002; Pearlin et al., 1990; Yates et al., 1999). In this study, we found that particular characteristics were associated with a change in burden, which could be related to the pandemic (such as the policy of nursing homes, where caregivers were not allowed to provide care as usual), but we cannot confirm this based on our cross‐sectional data. In return, increased objective and subjective burden was related to lower care‐related quality of life. We do not expect the normal progress of disease over a few months to be the main reason for these findings (Oldenkamp et al., 2016).

### 5.2 Limitations

Because the pandemic was unexpected, no data could be gathered before the pandemic. Therefore, participants were asked to recall their caregiving situation before the start of the pandemic. It is well possible that respondents may not remember all characteristics of this past caregiving situation completely accurately. However, because of the relatively short recall period and a topic that is familiar, relevant and probably central to the lives of respondents, we anticipate that the recall bias is limited. In addition, the effect of this bias is also likely to be random (McPhail & Haines, 2010). Furthermore, the way the questionnaire was administered may have resulted in potential sample selection bias. A comparison of the characteristics of our sample to respondents of the 2019 Informal Care survey of the National Institute of Social Research (de Boer et al., 2020) shows that our sample had similar characteristics with two exceptions: caregivers in our sample have been providing care for more years (7.2 compared to 5.4) and were more likely to provide care to their partner. Lastly, the questionnaire was experienced as long and at some points difficult, which may have led to selective attrition. Future research should take this into account.

### 5.3 Implications

Our findings have implications for policymakers aiming to target caregivers in times of crises and researchers aiming to evaluate the impact of a crisis or policies affecting caregivers. Our study indicates that informal caregivers are not a homogenous group and may experience different consequences from societal or personal crisis situations. Whether caregivers were affected and in what way depended on their gender, income, education, health, childcare responsibilities, duration of caregiving, travel distance to care recipient, needs of the care recipient and the social network of the care recipient. Although our study focuses on a rather extreme crisis situation, namely the COVID‐19 pandemic, there are many—and much more frequent—smaller crises over the course of the caregiving process, including those caused by influenza or norovirus outbreaks at nursing homes, unexpected events in competing roles of childcare and work or changes in health and social care provision.

## 6 CONCLUSION

In this paper, we found that the objective burden of informal caregivers on average did not change during the COVID‐19 pandemic. Caregivers are essential workers and generally sustained their practices. There was only a small increase in subjective burden. However, further analyses showed that there were considerable differences between informal caregivers, and that informal caregivers who changed the amount of time spent on caregiving were not necessarily the same as those who experienced changes in perceived strain. Therefore, the burden of informal caregiving is not unidimensional, and policymakers should tailor support policies to the different needs of caregivers. Finally, some of the disruptions due to the pandemic are also exemplary for smaller personal crises that may occur in the caregiving process. Future studies should look into the implications of such crises, their effects on caregivers, and best policies to support them in maintaining their valuable role.

## AUTHOR CONTRIBUTIONS

Leonarda Bremmers: collected the data (equal); contributed data or analysis tools (equal); performed the analysis (supporting). Pieter Bakx: conceived and designed the analysis (equal); contributed data or analysis tools (equal); performed the analysis (supporting); wrote the paper (supporting). Job van Exel: conceived and designed the analysis (supporting); collected the data (equal); contributed data or analysis tools (supporting). Marianne van Bochove: conceived and designed the analysis (supporting); contributed data or analysis tools (supporting); performed the analysis (supporting).

## CONFLICT OF INTEREST

The authors declare no conflict of interest.

## ETHICS STATEMENT

This study was approved by the Research Ethics Review Committee of Erasmus School of Health Policy & Management (IRB 20–16).

## Supporting information


Appendix S1.

## Data Availability

Participants have provided informed consent for use of their data by researchers from Erasmus University Rotterdam, therefore, data are not available.

## References

[hsc13975-bib-0001] Allen, J. , Uekusa, S. , & Alpass, F. M. (2022). Longitudinal cohort study of depression and anxiety among older informal caregivers following the initial COVID‐19 pandemic response in Aotearoa New Zealand. Journal of Aging and Health. 10.1177/08982643211052713 PMC9008474 35412393

[hsc13975-bib-0002] Bom, J. , Bakx, P. , Schut, F. , & Van Doorslaer, E. (2019). The impact of informal caregiving for older adults on the health of various types of caregivers: A systematic review. Gerontologist, 59(5), e629–e642. 10.1093/geront/gny137 30395200 PMC6850889

[hsc13975-bib-0003] Broese van Groenou, M. I. , & De Boer, A. (2016). Providing informal care in a changing society. European Journal of Ageing, 13(3), 271–279. 10.1007/s10433-016-0370-7 27610055 PMC4992501

[hsc13975-bib-0004] Brouwer, W. B. F. , Van Exel, N. J. A. , Van Gorp, B. , & Redekop, W. K. (2006). The CarerQol instrument: A new instrument to measure care‐related quality of life of informal caregivers for use in economic evaluations. Quality of Life Research, 15(6), 1005–1021. 10.1007/s11136-005-5994-6 16900281

[hsc13975-bib-0005] Budnick, A. , Hering, C. , Eggert, S. , Teubner, C. , Suhr, R. , Kuhlmey, A. , & Gellert, P. (2021). Informal caregivers during the COVID‐19 pandemic perceive additional burden: Findings from an ad‐hoc survey in Germany. BMC Health Services Research, 21(353), 353. 10.1186/s12913-021-06359-7 33863337 PMC8050992

[hsc13975-bib-0006] Chappell, N. L. , & Reid, R. C. (2002). Burden and well‐being among caregivers: Examining the distinction. Gerontologist, 42(6), 772–780. 10.1093/geront/42.6.772 12451158

[hsc13975-bib-0007] de Boer, A. , de Klerk, M. , Verbeek‐Oudijk, D. , & Plaisier, I. (2020). Blijvende bron van zorg . www.scp.nl

[hsc13975-bib-0008] de Sousa, L. R. T. , Sequeira, C. , Ferré‐Grau, C. , & Araújo, O. (2022). Impact of the COVID‐19 outbreak on the difficulties and burden experienced by family caregivers of older dependent persons. The Journal of Mental Health Training, Education and Practice, 17(4), 355–365. 10.1108/jmhtep-04-2021-0036

[hsc13975-bib-0009] Dutch Government . (2020). COVID‐19 measures . https://www.rijksoverheid.nl/actueel/nieuws/2020/05/28/persmoment‐27‐mei‐actuele‐routekaart‐coronamaatregelen

[hsc13975-bib-0010] Dutch Ministry of Health . (2020). Dagbesteding in coronatijd . https://www.rijksoverheid.nl/binaries/rijksoverheid/documenten/publicaties/2020/12/14/dagbesteding‐in‐coronatijd/Dagbesteding+in+coronatijd+.pdf

[hsc13975-bib-0011] Fletcher, J. R. (2020). Structuring unequal relations: Role trajectories in informal dementia care. Sociology of Health and Illness, 43(1), 65–81. 10.1111/1467-9566.13194 32997379

[hsc13975-bib-0012] Greaney, M. , Kunicki, Z. , Drohan, M. , & Cohen, S. (2020). The impact of COVID‐19 on informal caregivers' health behaviors. Innovation in Aging, 4(1), 959. 10.1093/geroni/igaa057.3505

[hsc13975-bib-0013] Hale, T. , Angrist, N. , Goldszmidt, R. , Kira, B. , Petherick, A. , Phillips, T. , Webster, S. , Cameron‐Blake, E. , Hallas, L. , Majumdar, S. , & Tatlow, H. (2021). A global panel database of pandemic policies (Oxford COVID‐19 Government Response Tracker). Nature Human Behaviour, 5(4), 529–538. 10.1038/s41562-021-01079-8 33686204

[hsc13975-bib-0014] Hoefman, R. J. , Van Exel, J. , & Brouwer, W. (2013). How to include informal care in economic evaluations. PharmacoEconomics, 31(12), 1105–1119. 10.1007/s40273-013-0104-z 24218135

[hsc13975-bib-0015] Hoefman, R. J. , Van Exel, J. , Rose, J. M. , Van De Wetering, E. J. , & Brouwer, W. B. F. (2014). A discrete choice experiment to obtain a tariff for valuing informal care situations measured with the CarerQol instrument. Medical Decision Making, 34(1), 84–96. 10.1177/0272989X13492013 23771881

[hsc13975-bib-0016] Hofstaetter, L. , Judd‐Lam, S. , & Cherrington, G. (2022). Informal care in Australia during the COVID‐19 pandemic. International Journal of Care and Caring, 6(1–2), 253–259. 10.1332/239788221x16216124420027

[hsc13975-bib-0017] Koopman, E. , Heemskerk, M. , van der Beek, A. J. , & Coenen, P. (2020). Factors associated with caregiver burden among adult (19–64 years) informal caregivers—An analysis from Dutch Municipal Health Service data. Health and Social Care in the Community, 28(5), 1578–1589. 10.1111/hsc.12982 32207221 PMC7496310

[hsc13975-bib-0018] Lafferty, A. , Phillips, D. , Dowling‐Hetherington, L. , Fahy, M. , Moloney, B. , Duffy, C. , Paul, G. , Fealy, G. , & Kroll, T. (2021). Colliding worlds: Family carers' experiences of balancing work and care in Ireland during the COVID‐19 pandemic. Health & Social Care in the Community, 30(3), 1133–1142. 10.1111/hsc.13365 33891356 PMC8251184

[hsc13975-bib-0019] Lightfoot, E. , Moone, R. , Suleiman, K. , Otis, J. , Kutzler, C. , Turck, K. , & Yun, H. (2021). Concerns of family caregivers during COVID‐19: The concerns of caregivers and the surprising silver linings concerns of family caregivers during COVID‐19. Journal of Gerontological Social Work, 64(6), 656–675. 10.1080/01634372.2021.1898512 33724169

[hsc13975-bib-0020] Lorenz‐Dant, K. , & Comas‐Herrera, A. (2021). The impacts of COVID‐19 on unpaid carers of adults with long‐term care needs and measures to address these impacts: A rapid review of evidence up to November 2020. Journal of Long‐Term Care, 124–153. 10.31389/jltc.76

[hsc13975-bib-0021] McPhail, S. , & Haines, T. (2010). Response shift, recall bias and their effect on measuring change in health‐related quality of life amongst older hospital patients. Health and Quality of Life Outcomes, 8, 1–9. 10.1186/1477-7525-8-65 20618978 PMC2912788

[hsc13975-bib-0022] Montgomery, A. R. J. V. , Gonyea, J. G. , & Hooyman, N. R. (1985). Caregiving and the experience of subjective and objective burden. Family Relations, 34(1), 19–26.

[hsc13975-bib-0023] Oldenkamp, M. , Hagedoorn, M. , Slaets, J. , Stolk, R. , Wittek, R. , & Smidt, N. (2016). Subjective burden among spousal and adult‐child informal caregivers of older adults: Results from a longitudinal cohort study. BMC Geriatrics, 16(1), 1–11. 10.1186/s12877-016-0387-y 27923347 PMC5142272

[hsc13975-bib-0024] Park, S. S. (2020). Caregivers' mental health and somatic symptoms during COVID‐19. The Journals of Gerontology: Series B, 76(4), e235–e240. 10.1093/geronb/gbaa121 PMC7454918 32738144

[hsc13975-bib-0025] Pavolini, E. , & Ranci, C. (2008). Restructuring the welfare state: Reforms in long‐term care in Western European countries. Journal of European Social Policy, 18(3), 246–259. 10.1177/0958928708091058

[hsc13975-bib-0026] Pearlin, L. I. , Mullan, J. T. , Semple, S. J. , & Skaff, M. M. (1990). Caregiving and the stress process: An overview of concepts and their measures. Gerontologist, 30(5), 583–594. 10.1093/geront/30.5.583 2276631

[hsc13975-bib-0027] Prins, M. , Willemse, B. , Van Der Velden, C. , & Pot, A. M. (2021). Involvement, worries and loneliness of family caregivers of people with dementia during the COVID‐19 visitor ban in long‐term care facilities. Geriatric Nursing, 42(6), 1474–1480. 10.1016/j.gerinurse.2021.10.002 34678687 PMC8526350

[hsc13975-bib-0028] Raiber, K. , & Verbakel, E. (2021). Are the gender gaps in informal caregiving intensity and burden closing due to the COVID‐19 pandemic? Evidence from the Netherlands. Gender, Work and Organization. 10.1111/gwao.12725 PMC8444913 34548767

[hsc13975-bib-0029] Rodrigues, R. , Simmons, C. , Schmidt, A. E. , & Steiber, N. (2021). Care in times of COVID‐19: The impact of the pandemic on informal caregiving in Austria. European Journal of Ageing, 18(2), 195–205. 10.1007/s10433-021-00611-z 33727905 PMC7952831

[hsc13975-bib-0030] Santini, S. , Socci, M. , Fabbietti, P. , Lamura, G. , & Teti, A. (2022). Factors worsening and mitigating the consequences of the COVID‐19 outbreak on the overall health of informal caregivers of older people with long‐term care needs living in Germany and in Italy. International Journal of Environmental Research and Public Health, 19(1694). 10.3390/ijerph19031694 PMC8835160 35162718

[hsc13975-bib-0031] De Boer, A. , Hoefman, R. , De Klerk, M. , Plaisier, I. , & De Roos, S. (2020). Beleidssignalement maatschappelijke gevolgen coronamaatregelen Mantelzorgers. The Hague: SCP.

[hsc13975-bib-0032] Smaling, H. J. A. , Tilburgs, B. , & Achterberg, W. P. (2022). The impact of social distancing due to the COVID‐19 pandemic on people with dementia, family carers and healthcare professionals: A qualitative study. International Journal of Environmental Research and Public Health, 19(519). 10.3390/ijerph19010519 PMC8744737 35010779

[hsc13975-bib-0033] Truskinovsky, Y. , Finlay, J. M. , & Kobayashi, L. C. (2022). Caregiving in a pandemic: COVID‐19 and the well‐being of family caregivers 55+ in the United States. Medical Care Research and Review. 10.1177/10775587211062405 35001714

[hsc13975-bib-0034] Tur‐sinai, A. , Bentur, N. , & Fabbietti, P. (2021). Impact of the outbreak of the COVID‐19 pandemic on formal and informal care of community‐dwelling older adults: Cross‐national clustering of empirical evidence from 23 countries. Sustainability, 13(7277). 10.3390/su13137277

[hsc13975-bib-0035] Van Exel, N. J. M. , Scholte op Reimer, W. J. M. , Brouwer, W. B. F. , van den Berg, B. , Koopmanschap, M. A. , & van den Bos, G. A. M. (2004). Instruments for assessing the burden of informal caregiving for stroke patients in clinical practice: A comparison of CSI, CRA, SCQ and self‐rated burden. Clinical Rehabilitation, 18(2), 203–214. 10.1191/0269215504cr723oa 15053130

[hsc13975-bib-0036] White, C . (2020). Caring from a distance: Using new and familiar means of keeping in touch with family and friends in care homes during COVID‐19 . https://ltccovid.org/2020/07/24/next‐ltccovid‐webinar‐the‐impact‐of‐the‐covid‐19‐pandemic‐on‐unpaid‐carers‐3rd‐august‐2pm‐gmt/

[hsc13975-bib-0037] Yates, M. E. , Tennstedt, S. , & Chang, B. H. (1999). Contributors to and mediators of psychological well‐being for informal caregivers. Journals of Gerontology: Series B Psychological Sciences and Social Sciences, 54(1), 12–22. 10.1093/geronb/54B.1.P12 9934391

[hsc13975-bib-0038] Zwar, L. , König, H.‐H. , & Hajek, A. (2022). gender differences in mental health, quality of life, and caregiver burden among informal caregivers during the second wave of the COVID‐19 pandemic in Germany: A representative population‐based study. Gerontology, 1–14. 10.1159/000523846 PMC9148891 35390788

