# Supplementary material for: Informal care in times of a public health crisis: Objective burden, subjective burden and quality of life of caregivers in the Netherlands during the COVID‐19 pandemic
Source: Health Soc Care Community. 2022 Sep 6:10.1111/hsc.13975. Online ahead of print. doi: 10.1111/hsc.13975 (PMC9538241; doi:10.1111/hsc.13975)
Supplement: Supplementary file 1 — Appendix S1. [file HSC-9999-0-s001.docx]

1. **Appendix**
   1. **Explanation of variables^1^**

| **Variable** | **Question in survey** | **Answer categories** | **Adjusted for use in this paper** |
| --- | --- | --- | --- |
| **Outcome variables** | | | |
| Objective burden | How many hours did you spend on household tasks in the last week?  These are tasks that you do extra because the person to whom you give help cannot do this due to health problems.  In the past week, did you provide more or fewer hours of help with household tasks than before the start of the corona crisis?  We asked the same questions for ‘nursing’, ‘practical support’, and ‘emotional support’. | ___ number of hours per week   - I gave about the same amount of hours - I gave ___ hours more than before - I gave ___ hours less than before | Hours per sub domain were maximized at 18 hours a day, 7 days a week (126 hours). Then, these hours were summed up for during the pandemic (t=1). The change was directly asked. Furthermore, the change was subtracted from or summed up with that amount to calculate the objective burden before the pandemic (t=0). Again, the total was capped at 126 hours. If participants filled out the question so that the hours before the pandemic were less than zero, the value was set to 0. |
| Subjective burden | How burdensome do you find informal care for the person to whom you are currently providing help?  How burdensome did you find the informal care for the person you are providing help to in the month before the start of the corona crisis? | Answers were asked on a scale from 0-10, with 0 being “not burdensome at all” and 10 being “most burdensome imaginable”. | The change was calculated by subtracting the score on the first question from the second question. |
| Care-related quality of life (CarerQol (Brouwer et al., 2006)) | I have ___ satisfaction in performing my care duties  I have ___ relationship problems with her / him (e.g. she/he is very demanding, has started to behave differently, we have communication problems)  I have ___ problems with my own mental health (e.g. stress, anxiety, sadness, worry about the future)  I have ___ problems combining my daily activities with my care tasks (e.g. work, study, housekeeping, family and leisure activities)  I have ___ financial problems with my care duties  I have ___ support (from family/friends/neighbors/acquaintances) in performing my care duties, if necessary  I have ___ problems with my own physical health  (e.g. more often sick, fatigue, physical overload) | All sub-questions have the same three answer categories: no/somewhat/a lot | Weights provided by Hoefman et al. (2014) were used for the items. |
| **Caregiver characteristics** | | | |
| Woman | What is your gender? | Man, woman | NA |
| Age | What is your age? | ___ years old | NA |
| Level of education | What is your highest attained education? | - LO (lagere school, LAVO, VGLO)^2^ - LBO (LBO, LTS, ITO, LEAO, Huishoudschool, LLO)^2^ - MAO (MAVO, IVO, MULO, ULO, 3jr HBS, 3jr VWO, 3jr VHMO)^2^ - MBO (MTS, UTS, MEAO)^2^ - HAO (HAVO, VWO, Atheneum, Gymnasium, NMS, HBS, Lyceum)^2^ - HBO (HTS, HEAO, Wetensch. kand., Univers. onderwijs kand.)^2^ - WO (Universitair onderwijs, Doctoraalopleiding, TH)^2^ | We aggregated education level into three categories.  Low: primary education or practical secondary education (the first three categories)  Middle: other secondary education or practical tertiary education (the third and fourth category)  High: other levels of tertiary and further education (the last two categories) |
| Ability to make ends meet | When you think of your household's total income per month, how well can your household currently make ends meet? | Answer categories: very easy, somewhat easy, somewhat difficult, very difficult. | NA |
| Psychological health^3^ | Indicate on the scale below what your psychological well-being is at the moment. A 0 stands for the worst imaginable psychological well-being and 10 for the best imaginable psychological well-being.  Please indicate on the scale below what your psychological well-being was like before the start of the corona crisis. A 0 stands for the worst imaginable psychological well-being and 10 for the best imaginable psychological well-being. | Both from 0-10 | The change was calculated by subtracting the score on the first question from the second question. |
| Physical health^3^ | Rate your health at this point. The '0' stands for the worst health you can imagine, the '10' for the best health you can imagine.  Before the start of the corona crisis, rate your health. The '0' stands for the worst health you can imagine, the '10' for the best health you can imagine. | Both from 0-10 | The change was calculated by subtracting the score on the first question from the second question. |
| Work status | What is your current work situation?  If several options apply (for example, if you are working and studying part-time), select the activity that takes up the most time. | - Full-time or part-time - Retired - Unable to work, incapacitated for work - Student, high school student - Not working, looking for a job - Housewife/houseman | Answers were aggregated into three categories.  Working: the first answer category  Retired: the second answer category  Not working: all other answer categories |
| Hours employment | How many hours per week do you currently work?  Only count the hours for which you are paid.  How many hours per week did you work in a paid job before the start of the corona crisis? Only count the hours for which you were paid. | ___ hours | The change was calculated by subtracting the score on the first question from the second question. |
| Childcare responsibilities | Heeft u thuiswonende kinderen? | - No   Yes, my youngest child's age is ____ years old | Answers were transformed in two categories, where people whose youngest child at home was 18 years or older were counted as ‘no childcare repsonsibilities’. |
| **Relationship with care recipient** | | | |
| Relationship | What is the relationship of the person you are helping with you? She or he is my ... | - Partner - Mother or father - Mother-in-law or father-in-law - Daughter or son - Sister or brother - Other family member - Friend or acquaintance - Neighbor or neighbor - Someone else, namely ___ | We aggregated relationship in four categories.  Partner  Parent: including in-law  Other family member: all other family members  Friends and other: all other categories |
| Duration of care | How long have you been providing this help?  If less than one year, enter 0 for years. | ___ years and ___ months | Transformed to years |
| Living situation | Does the person to whom you provide assistance live in a care or nursing home, an institution for the mentally handicapped or another care institution? | - No, she/he lives with me - No, she/he lives independently (alone or with others) - Yes, she/he lives in a care or nursing home - Yes, she/he lives in an institution for the mentally handicapped or another care institution - Yes, she/he lives in another healthcare institution - Other, namely ___ | Answers were aggregated into three categories.  Same home: only the first answer category  Other home: only the second answer category  Institution: the third up to the fifth answer category  11 participants filled out “other” and were placed in categories based on their answer in the following open question. |
| Travel distance | How many minutes on average does it take you to get to the person you are helping?  Please provide the average time for a one-way trip from your home to her / his home | ___ minutes | NA |
| **Care recipient characteristics** | | | |
| Social network of recipient | If the person you are giving help to needs someone, how many people could she/he ask for?  Please do not include yourself | ___ people | NA |

^1^Translated here from Dutch to English.

^2^Due to the specific Dutch situation, these questions/answers remain untranslated.

^3^These questions were also asked about the care recipient

- 1. **Additional analyses**
     1. **Additional analyses: extra control variables**

We conducted the extra analyses that Referee 1 requested and report these below. In these extra analyses, we include changes in objective burden as a predictor for changes in subjective burden, and levels and changes of objective and subjective burden as predictor for care-related quality of life during COVID-19. For the change in subjective burden, we find a small positive relation between a change in objective burden and a change in subjective burden, while the other coefficients in the model remain largely the same (see table 1 below). For care-related quality of life, the coefficients the sign does not change and the magnitude is very similar compared to the main analysis. In the version with additional control variables, four coefficients are significantly different from zero that were not significant before. Furthermore, we found that the newly added predictors (levels of and changes in objective and subjective burden) are strongly associated with the outcome.

**Table 1.** OLS regressions with extra control variables

|  | Analysis with  additional controls | | Main analysis | | Analysis with  additional controls | | Main analysis | |
| --- | --- | --- | --- | --- | --- | --- | --- | --- |
|  | (1)  ∆ Subjective burden | | (2)  ∆ Subjective burden | | (3)  Care-related  quality of life (t=1) | | (4)  Care-related  quality of life (t=1) | |
|  | β | (SE) | β | (SE) | β | (SE) | β | (SE) |
| **Care recipient care need** |  |  |  |  |  |  |  |  |
| Psychological health of recipient (t=0) | -0.007 | (0.027) | -0.002 | (0.027) | 0.358 | (0.282) | 0.907*** | (0.308) |
| ∆ Psychological health of recipient | -0.108*** | (0.035) | -0.107*** | (0.035) | -0.315 | (0.364) | -0.027 | (0.401) |
| Physical health of recipient (t=0) | -0.017 | (0.029) | -0.016 | (0.029) | -0.665** | (0.300) | -0.463 | (0.332) |
| ∆ Physical health of recipient | -0.176*** | (0.038) | -0.178*** | (0.038) | -0.454 | (0.400) | -0.316 | (0.439) |
| **Caregiver characteristics** |  |  |  |  |  |  |  |  |
| Woman (=1) | -0.314*** | (0.096) | 0.319*** | (0.096) | 0.475 | (1.018) | -1.482 | (1.117) |
| Age caregiver | 0.005 | (0.004) | 0.005 | (0.004) | 0.118*** | (0.043) | 0.147*** | (0.047) |
| *Education (ref=low)* |  |  |  |  |  |  |  |  |
| Middle | -0.049 | (0.122) | -0.045 | (0.122) | 1.593 | (1.277) | 2.941** | (1.414) |
| High | 0.015 | (0.131) | 0.022 | (0.131) | -0.604 | (1.370) | -0.007 | (1.518) |
| *Ability to make ends meet (ref=fairly easily)* |  |  |  |  |  |  |  |  |
| With great difficulty | 0.116 | (0.180) | 0.107 | (0.180) | -8.393*** | (1.909) | -12.407*** | (2.086) |
| With some difficulty | 0.244** | (0.099) | 0.232** | (0.099) | -1.491 | (1.050) | -3.226*** | (1.151) |
| Easily | 0.173 | (0.136) | 0.167 | (0.136) | 0.271 | (1.421) | 1.427 | (1.574) |
| Psychological health of caregiver (t=0) | -0.058 | (0.036) | -0.058 | (0.036) | 1.735*** | (0.381) | 2.435*** | (0.420) |
| ∆ Psychological health of caregiver | -0.137*** | (0.040) | -0.136*** | (0.040) | 1.563*** | (0.425) | 1.842*** | (0.469) |
| Physical health of caregiver (t=0) | 0.103*** | (0.036) | 0.103*** | (0.037) | 1.541*** | (0.383) | 1.400*** | (0.424) |
| ∆ Physical health of caregiver | 0.056 | (0.052) | 0.059 | (0.052) | 1.343** | (0.541) | 1.887*** | (0.598) |
| *Work status (ref=working)* |  |  |  |  |  |  |  |  |
| Not working | 0.063 | (0.198) | 0.048 | (0.198) | -3.029 | (2.073) | -3.218 | (2.291) |
| Retired | -0.010 | (0.207) | -0.026 | (0.207) | -1.979 | (2.181) | -3.785 | (2.401) |
| Hours employment (t=0) | 0.002 | (0.006) | 0.002 | (0.006) | -0.039 | (0.059) | -0.108* | (0.065) |
| ∆ Hours employment | -0.012 | (0.008) | -0.013 | (0.009) | -0.082 | (0.089) | -0.100 | (0.099) |
| Childcare responsibilities (t=1) | 0.215* | (0.117) | 0.211* | (0.118) | -3.831*** | (1.240) | -6.298*** | (1.362) |
| *Relationship (ref=partner)* |  |  |  |  |  |  |  |  |
| Parent | -0.039 | (0.165) | -0.038 | (0.165) | 1.420 | (1.744) | 2.277 | (1.915) |
| Other family member | -0.104 | (0.171) | -0.094 | (0.171) | 1.264 | (1.794) | 1.398 | (1.982) |
| Friends and other | -0.109 | (0.190) | -0.095 | (0.190) | 1.395 | (1.995) | 3.481 | (2.199) |
| Duration of care | 0.013** | (0.006) | 0.014** | (0.006) | -0.085 | (0.063) | -0.143** | (0.069) |
| *Living situation (ref=in same home)* |  |  |  |  |  |  |  |  |
| Other private home | 0.207 | (0.154) | 0.189 | (0.154) | -0.720 | (1.640) | 1.996 | (1.782) |
| An institution | 0.207 | (0.172) | 0.153 | (0.170) | -2.580 | (1.817) | -1.005 | (1.974) |
| Travel distance | -0.001 | (0.001) | -0.001 | (0.001) | -0.030** | (0.013) | -0.043*** | (0.014) |
| **Social network of recipient** | 0.034 | (0.031) | 0.033 | (0.031) | 1.455*** | (0.322) | 1.636*** | (0.356) |
| **Extra mediator variables** |  |  |  |  |  |  |  |  |
| Objective burden (t=0) |  |  |  |  | -0.054*** | (0.019) |  |  |
| ∆ Objective burden | 0.017** | (0.007) |  |  | -0.138* | (0.080) |  |  |
| Subjective burden (t=0) |  |  |  |  | -2.846*** | (0.208) |  |  |
| ∆ Subjective burden |  |  |  |  | -2.008*** | (0.352) |  |  |
|  |  |  |  |  |  |  |  |  |
| Constant | -0.532 | (0.413) | -0.544 | (0.414) | 63.728*** | (4.729) | 36.994*** | (4.793) |
| Observations | 965 |  | 965 |  | 965 |  | 965 |  |
| R-squared | 0.116 |  | 0.111 |  | 0.464 |  | 0.336 |  |

- - 1. **Additional analyses: multinomial logistic regressions**

In the paper we use an ordinary least squares regression. Alternatively, a multinomial logistic regression could be performed for the outcomes of objective and subjective burden. This means that the continuous outcomes were divided into three categories: a negative change in (either objective or subjective) burden, a positive change in (either objective or subjective burden), and no change. The latter category was used as a reference group. For all independent variables the relative risk ratio (RRR) is presented. The RRR represents the predicted risk of falling in this category relative to the risk of falling in the reference category (here: no change in burden) for each one unit increase of the independent variable (see tables 2 and 3 below). A RRR of close to 1 implies no increased risk for one group over the other to experience an increase or a decrease in burden compared to experiencing no change in burden. A RRR of lower than 1 means a reduced risk, a RRR of higher than 1 means an increased risk. These analyses show the heterogeneity behind the average effects of a variable but this comes at the cost of not being able to account for variation in the intensity of the change. For example, the main analysis (column 3 of Table 2) shows that there was no statistically significant difference between women and men in whether they experienced a change in objective burden. However, the additional analyses (columns 1 and 2 of Table 2) show that women were more likely to experience no change in objective burden, while men were more likely to experience either an increase or a decrease in objective burden.

**Table 2.** Multinomial logistic regression with outcome categories less objective burden, more objective burden and same objective burden as before the pandemic. The latter is the reference group.

|  | Multinomial logit | | | | Main analysis | |
| --- | --- | --- | --- | --- | --- | --- |
|  | (1)  Less objective burden | | (2)  More objective burden | | (3)  △ Objective burden | |
|  | RRR | (SE) | RRR | (SE) | β | (SE) |
| **Care recipient care need** |  |  |  |  |  |  |
| Psychological health of recipient (t=0) | 0.820*** | (0.053) | 1.022 | (0.065) | 0.295** | (0.117) |
| ∆ Psychological health of recipient | 0.703*** | (0.060) | 0.735*** | (0.059) | 0.001 | (0.152) |
| Physical health of recipient (t=0) | 0.948 | (0.066) | 1.002 | (0.066) | 0.086 | (0.126) |
| ∆ Physical health of recipient | 0.937 | (0.085) | 0.899 | (0.075) | -0.145 | (0.166) |
| **Caregiver characteristics** |  |  |  |  |  |  |
| Woman (=1) | 0.624** | (0.149) | 0.638** | (0.144) | 0.289 | (0.423) |
| Age caregiver | 1.014 | (0.010) | 1.009 | (0.010) | -0.012 | (0.018) |
| *Education (ref=low)* |  |  |  |  |  |  |
| Middle | 0.982 | (0.298) | 1.132 | (0.332) | 0.182 | (0.536) |
| High | 1.113 | (0.358) | 1.524 | (0.467) | 0.390 | (0.575) |
| *Ability to make ends meet (ref=fairly easily)* |  |  |  |  |  |  |
| With great difficulty | 0.637 | (0.306) | 1.100 | (0.429) | -0.500 | (0.790) |
| With some difficulty | 0.967 | (0.231) | 0.853 | (0.196) | -0.690 | (0.436) |
| Easily | 0.834 | (0.287) | 0.999 | (0.313) | -0.360 | (0.596) |
| Psychological health of caregiver (t=0) | 1.022 | (0.088) | 0.899 | (0.073) | -0.000 | (0.159) |
| ∆ Psychological health of caregiver | 0.907 | (0.093) | 0.852* | (0.074) | 0.042 | (0.178) |
| Physical health of caregiver (t=0) | 0.929 | (0.081) | 0.990 | (0.081) | 0.007 | (0.160) |
| ∆ Physical health of caregiver | 0.910 | (0.116) | 0.885 | (0.099) | 0.178 | (0.227) |
| *Work status (ref=working)* |  |  |  |  |  |  |
| Not working | 1.192 | (0.584) | 0.858 | (0.375) | -0.890 | (0.868) |
| Retired | 1.020 | (0.520) | 0.831 | (0.387) | -0.920 | (0.909) |
| Hours employment (t=0) | 0.999 | (0.014) | 0.989 | (0.013) | -0.012 | (0.025) |
| ∆ Hours employment | 0.998 | (0.022) | 0.961** | (0.017) | -0.051 | (0.037) |
| Childcare responsibilities (t=1) | 1.617* | (0.461) | 1.276 | (0.344) | -0.254 | (0.516) |
| *Relationship (ref=partner)* |  |  |  |  |  |  |
| Parent | 1.135 | (0.499) | 1.039 | (0.398) | 0.072 | (0.725) |
| Other family member | 0.696 | (0.320) | 0.770 | (0.303) | 0.601 | (0.751) |
| Friends and other | 0.727 | (0.353) | 0.462* | (0.213) | 0.785 | (0.833) |
| Duration of care | 1.025* | (0.015) | 1.028** | (0.013) | 0.038 | (0.026) |
| *Living situation (ref=in same home)* |  |  |  |  |  |  |
| Other private home | 2.742** | (1.227) | 1.849* | (0.661) | -1.095 | (0.675) |
| An institution | 9.328*** | (4.210) | 2.164* | (0.881) | -3.108*** | (0.747) |
| Travel distance | 0.999 | (0.003) | 0.993 | (0.004) | 0.001 | (0.005) |
| **Social network of recipient** | 1.021 | (0.076) | 1.036 | (0.073) | -0.060 | (0.135) |
|  |  |  |  |  |  |  |
| Constant | 0.186 | (0.194) | 0.131** | (0.126) | -0.666 | (1.815) |
| Observations | 965 |  | 965 |  | 965 |  |

**Table 3.** Multinomial logistic regression with outcome categories less subjective burden, more subjective burden and same subjective burden as before the pandemic. The latter is the reference group.

|  | Multinomial logit | | | | Main analysis | |
| --- | --- | --- | --- | --- | --- | --- |
|  | (1)  Less subjective burden | | (2)  More objective burden | | (3)  △ Subjective burden | |
|  | RRR | (SE) | RRR | (SE) | β | (SE) |
| **Care recipient care need** |  |  |  |  |  |  |
| Psychological health of recipient (t=0) | 1.003 | (0.077) | 0.990 | (0.050) | -0.002 | (0.027) |
| ∆ Psychological health of recipient | 0.943 | (0.087) | 0.823*** | (0.054) | -0.107*** | (0.035) |
| Physical health of recipient (t=0) | 0.994 | (0.081) | 0.964 | (0.052) | -0.016 | (0.029) |
| ∆ Physical health of recipient | 0.996 | (0.101) | 0.811*** | (0.057) | -0.178*** | (0.038) |
| **Caregiver characteristics** |  |  |  |  |  |  |
| Woman (=1) | 1.587* | (0.415) | 0.708* | (0.129) | 0.319*** | (0.096) |
| Age caregiver | 0.969*** | (0.010) | 0.991 | (0.007) | 0.005 | (0.004) |
| *Education (ref=low)* |  |  |  |  |  |  |
| Middle | 0.903 | (0.338) | 0.821 | (0.192) | -0.045 | (0.122) |
| High | 1.537 | (0.591) | 1.463 | (0.356) | 0.022 | (0.131) |
| *Ability to make ends meet (ref=fairly easily)* |  |  |  |  |  |  |
| With great difficulty | 1.034 | (0.494) | 1.827* | (0.592) | 0.107 | (0.180) |
| With some difficulty | 0.788 | (0.220) | 1.331 | (0.247) | 0.232** | (0.099) |
| Easily | 1.384 | (0.510) | 1.238 | (0.324) | 0.167 | (0.136) |
| Psychological health of caregiver (t=0) | 0.885 | (0.086) | 0.834*** | (0.056) | -0.058 | (0.036) |
| ∆ Psychological health of caregiver | 0.964 | (0.105) | 0.802*** | (0.060) | -0.136*** | (0.040) |
| Physical health of caregiver (t=0) | 0.962 | (0.097) | 1.153** | (0.080) | 0.103*** | (0.037) |
| ∆ Physical health of caregiver | 0.875 | (0.113) | 1.020 | (0.098) | 0.059 | (0.052) |
| *Work status (ref=working)* |  |  |  |  |  |  |
| Not working | 1.248 | (0.674) | 1.676 | (0.600) | 0.048 | (0.198) |
| Retired | 0.708 | (0.434) | 0.976 | (0.381) | -0.026 | (0.207) |
| Hours employment (t=0) | 0.986 | (0.015) | 1.009 | (0.010) | 0.002 | (0.006) |
| ∆ Hours employment | 1.001 | (0.022) | 1.006 | (0.015) | -0.013 | (0.009) |
| Childcare responsibilities (t=1) | 1.874** | (0.536) | 1.584** | (0.331) | 0.211* | (0.118) |
| *Relationship (ref=partner)* |  |  |  |  |  |  |
| Parent | 1.011 | (0.461) | 1.030 | (0.319) | -0.038 | (0.165) |
| Other family member | 1.409 | (0.667) | 1.186 | (0.379) | -0.094 | (0.171) |
| Friends and other | 0.902 | (0.478) | 1.014 | (0.363) | -0.095 | (0.190) |
| Duration of care | 0.951** | (0.022) | 1.003 | (0.011) | 0.014** | (0.006) |
| *Living situation (ref=in same home)* |  |  |  |  |  |  |
| Other private home | 0.813 | (0.333) | 1.060 | (0.302) | 0.189 | (0.154) |
| An institution | 1.989 | (0.862) | 1.917** | (0.599) | 0.153 | (0.170) |
| Travel distance | 1.004 | (0.003) | 1.001 | (0.002) | -0.001 | (0.001) |
| **Social network of recipient** | 0.974 | (0.085) | 1.065 | (0.061) | 0.033 | (0.031) |
|  |  |  |  |  |  |  |
| Constant | 2.346 | (2.514) | 0.438 | (0.339) | -0.544 | (0.414) |
| Observations | 965 |  | 965 |  | 965 |  |
